# Response of cattle with clinical osteochondrosis to mineral supplementation

**DOI:** 10.4102/ojvr.v84i1.1365

**Published:** 2017-02-24

**Authors:** Gerjan van der Veen, Geoffrey T. Fosgate, Frederick K. Botha, Heinz H. Meissner, Lubbe Jacobs, Leon Prozesky

**Affiliations:** 1 Department of Paraclinical Sciences, University of Pretoria, South Africa; 2 Department of Production Animal Studies, University of Pretoria, South Africa; 3 Lubern Animal Feeds, Hartswater, South Africa

## Abstract

Since 1982, farmers in the North West province and other parts of South Africa have noticed an increase in the incidence of lameness in cattle. Macro- and microscopical lesions of joints resembled osteochondrosis. Pre-trial data indicated that cattle with osteochondrotic lesions recovered almost completely when fed a supplement containing bio-available micro- and macrominerals of high quality. In the present trial, 43 clinically affected cattle of varying ages (1–5 years) and sexes were randomly divided into three groups. Each group was fed the same commercial supplement base with differing micro- and macromineral concentrations to determine the effect of mineral concentrations on the recovery from osteochondrosis. Both supplements 1 and 2 contained 25% of the recommended National Research Council (NRC) mineral values. Additional phosphate was added to supplement 2. Supplement 3, containing 80% of the NRC mineral values, was used as the control. Results from all three groups indicated no recovery from osteochondrosis. Urine pH of a small sample of the test cattle showed aciduria (pH < 6). Supplement analysis revealed addition of ammonium sulphate that contributed sulphate and nitrogen to the supplement. Supplementary dietary cation anion difference (DCAD) values were negative at -411 mEq/kg, -466 mEq/kg and -467 mEq/kg for supplements 1, 2 and 3, respectively, whereas the pre-trial supplement was calculated at +19.87 mEq/kg. It was hypothesised that feeding a low (negative) DCAD diet will predispose growing cattle to the development of osteochondrosis or exacerbate subclinical or clinical osteochondrosis in cattle.

## Introduction

‘Osteochondrosis’ is a broad term pertaining to a group of lesions associated with the persistence of growth cartilage in the epiphyseal or metaphyseal growth plate as a result of failure of endochondral ossification (Laverty & Girard 2013; Olsson 1987; Olsson & Reiland 1977; Ytrehus, Carlson & Ekman 2007). König first introduced the term in 1888 to describe a pathological condition of the articular cartilage that leads to the formation of loose bodies in the joint (Ytrehus et al. 2007).

Different aetiological factors for osteochondrosis have been proposed. Rapid growth has been described as one of the main contributing factors predisposing to the development of osteochondrosis (Ekman & Carlson 1998; Olsson & Reiland 1977; Reiland 1977). Osteochondrosis has been described in pigs (Grøndalen 1974), dogs (Trostel, McLaughlin & Pool 2002), newborn lambs (Corbellini et al. 1991), poultry (Whitehead 1997), turkeys (Poulos 1977), horses (Jeffcott 1991), cattle (Trostle et al. 1998), cats (Ralphs 2005) and rats (Kato & Onodera 1984).

Since 1982, farmers in the North West province and other parts of South Africa have noticed an increase in the incidence of lameness in cattle. Clinical signs included varying degrees of lameness and peri-articular swelling, especially of the stifle joints. Macro- and microscopical lesions of joints resembled osteochondrosis. The initial aetiological factor thought to predispose to the development of osteochondrosis was that of a mineral deficiency, particularly phosphate. The North West province of South Africa is known for its mineral-related pathologic conditions such as osteomalacia, botulism (Theiler, Green & Du Toit 1928) and Vryburg hepatosis (Elsenbroek & Neser 2002; Neser et al. 1997). A survey conducted in 2004 indicated that several similar factors such as the breeding season, age, sex, anatomical conformation, nutritional supplementation and management were all commonly found throughout the affected geographical region. It was concluded that osteochondrosis is not the result of a single factor, but a multifactorial problem (Prozesky et al. 2016).

The most common joints of cattle affected by osteochondrosis are the shoulder, elbow and stifle joint (Hill, Sutton & Thompson 1998; Jensen et al. 1981; Reiland et al. 1977; Trostle et al. 1997; Weisbrode et al. 1982). Peri-articular swelling as a result of inflammation (Trostle et al. 1998) can be noticed and is most evident in the elbow and stifle joints. Osteochondrosis of the stifle joint alters the anatomical conformation of the hind leg. The normal angular conformation is lost as the affected hind leg straightens as the lesion progresses in severity (Hill et al. 1998). Severely affected cattle adapt a sawhorse stance to reduce the load on affected joints.

Several immature Brahman cattle of both sexes (exact number unknown) suffering from varying degrees of lameness and peri-articular swelling (clinical osteochondrosis) were included in a feeding trial (henceforth referred to as the pre-trial) during December 2012 at Onderstepoort. The purpose of the trial was to determine if cattle with clinical osteochondrosis would benefit from additional mineral supplementation. The supplement was fed for a period of 3 weeks and contained above normal levels of bio-available micro- and macrominerals. The clinically affected cattle responded positively with a marked decrease in the severity of lameness and size of the peri-articular swelling (unpublished data). Results of the pre-trial emphasised the importance of determining the concentration of minerals necessary to be included in a supplement that would benefit cattle suffering from clinical osteochondrosis. This study was conducted to investigate the required mineral levels that would promote clinical improvement of cattle with clinical osteochondrosis.

## Material and methods

Forty-three clinically affected Brahman cattle of varying ages and sexes, originating from the same geographical area, were included in the feeding trial. Animals were weighed and clinically evaluated at the start of the trial.

The trial supplements were formulated to have the same basic ingredients and to only vary in micro- and macro-mineral concentrations. The three supplements were as follows:

- Supplement 1 contained 25% of the recommended NRC (2001) mineral values. No additional phosphate was added to the supplement.
- Supplement 2 contained 25% of the recommended NRC (2001) mineral values with the exception of added mono-ammonium phosphate (MAP) to an equivalent level to that of supplement 3.
- Supplement 3 contained 80% of the recommended NRC (2001) mineral levels and was used as the control diet.

For each supplement, the same mineral premix base, Arthrocure B (ANH^®^, pers. comm., 2013), was included to ensure comparable standard and quality of minerals. The bioavailability of both micro- and macrominerals included in the Arthrocure premix is high (composition not shown because of confidentiality implications). The absorption percentage of the phosphate source used (MAP) in supplements 2 and 3 according to the National Research Council (2001) is 80%. Ingredients and amounts used to formulate the trial supplementary feeds are provided in Table 1.

**TABLE 1:** List of ingredients and amounts used to formulate the respective trial supplements.

Ingredients	Supplementary feed 1		Supplementary feed 2		Supplementary feed 3	
		
	As is %	Mix (kg)	As is %	Mix (kg)	As is %	Mix (kg)
Salt	25.00	250	25.0	250	25.00	250.0
Bran 15%	15.00	150	12.5	125	12.50	125.0
Hominy chop	13.00	130	12.4	124	12.40	124.0
Peanut shells	12.70	127	7.8	78	7.00	70.0
Urea	12.00	120	11.0	110	11.00	110.0
Molasses	12.00	120	12.0	120	11.95	119.5
Sunflower O/C 38%	6.00	60	6.0	60	6.00	60.0
Ammonium sulphate	3.00	30	3.0	30	3.00	30.0
Limestone	1.00	10	5.0	50	5.00	50.0
Mono-ammonium phosphate (MAP)	0.00	0	5.0	50	5.00	50.0
DSM Arthrocure B	0.30	3	0.3	3	1.15	11.5

**Total**	**100**	**1000**	**100**	**1000**	**100**	**1000**

This table presents the formulation of the three different supplementary feeds used during the trial. The original supplement formulation did not include ammonium sulphate, and it was only after analysis that the inclusion thereof became known. Both the as is % and kilogram (kg) amounts of the respective ingredients for each supplement are presented.

Cattle had *ad lib* access to baled *Eragrostis teff* hay for the total duration of the trial. Supplementary feed was collected and weighed on a weekly basis to determine the average individual consumption (grams) per day. The cattle were fed for a total period of 12 weeks.

**FIGURE 1:**
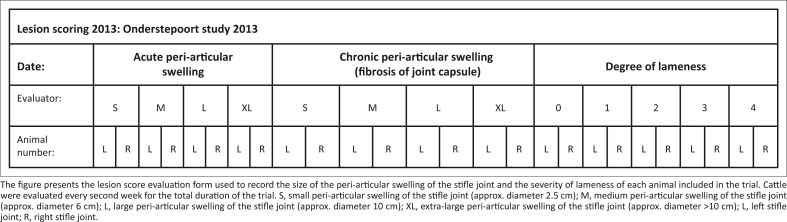
Visual lesion evaluation form.

The cattle were evaluated clinically every second week for the total duration of the trial. Three independent observers each individually recorded clinical signs to limit any biased decisions. Visual evaluation included grading of both the size of the peri-articular swelling and the degree of lameness. Acute peri-articular swelling has a pronounced bulging appearance, whereas chronic peri-articular swelling has a more flattened appearance because of thickening of the joint capsule that depresses the swelling. The chronicity, size and location of each peri-articular swelling were recorded in a format similar to Figure 1.

Lameness was graded on a scale of severity as shown in Table 2.

**TABLE 2:** Grading score for lameness.

Lameness score	Score definition
0	No clinical signs of lameness observed
1	Conformational changes noticed and slight lameness observed
2	Moderate lameness observed
3	Severely affected but still weight-bearing
4	Severely affected with very little to non-weight-bearing

This table presents the criteria used to determine the severity of lameness. Each hind leg of the animal was scored individually.

### Statistical analysis

An overall disability score was calculated as the sum of the scores for each clinical category (lameness, acute and fibrosis) over both hind legs (left and right). The change in each clinical category score was calculated by subtracting off the baseline from all subsequent values (forming ‘delta’ scores). Delta scores were assessed for normality by calculating descriptive statistics, plotting histograms and performing the Anderson-Darling test in available software (MINITAB Statistical Software, Release 13.32, Minitab Inc, State College, PA, USA). Categorical data were described using proportions and 95% mid-P exact confidence intervals (CI) and compared among diets using chi-square tests. Categorical data analysis was performed using available freeware (Epi Info, version 6.04, CDC, Atlanta, GA, USA). Delta scores were descriptively presented as medians and ranges and transformed by ranking prior to statistical analysis. Delta scores were compared among diet groups using linear mixed models that included animal as a random effect and diet as a fixed effect. Unless stated otherwise, statistical analyses were performed using commercially available software (IBM SPSS Statistics Version 22, International Business Machines Corp., Armonk, NY, USA) and results interpreted at the 5% level of significance.

Forty-three cattle with clinical osteochondrosis were randomly divided into three groups. Groups were housed in separate camps, and each camp was equipped with a water trough (municipal water), hayrack and two Taltec feed troughs that provided sufficient feeding space.

### Clinical examination and urinalysis

The clinical examination was done to investigate any possible causes for the negative results obtained during the trial. Results obtained from the clinical examination were used for differential diagnoses only. Cattle sample size was not statistically calculated as the examination was only done after the negative results were obtained and was for interest’ sake only.

Urine was collected from cattle that urinated voluntarily in the crush. The same animals from which urine was collected were clinically examined. Observers used a pole with a cup attached at the end to collect the free flow urine sample. Collected urine was analysed immediately with the Combur^9^Test^®^ (Roche) urine dipstick.

## Results

### Initial weight, supplement and phosphate intake

Statistical analysis indicated that there was no significant difference between the starting weights of the respective groups. The average weight of groups 1, 2 and 3 was 310 kg, 290 kg and 267 kg, respectively. Supplement intake varied between the groups, with the average collective daily supplement intakes for groups 1, 2 and 3 being 370 g, 255 g and 292 g, respectively. Daily supplement intake calculated as grams consumed per kg live weight was 1.19 g, 0.88 g and 0.91 g per kg live weight, respectively, for groups 1, 2 and 3.

The calculated average daily phosphate intake per animal differed between the groups. Consumed values are presented in Table 3.

**TABLE 3:** Calculated average daily phosphate intake of respective sub-groups.

Supplement and sub-group	Average starting weight of the cattle (kg)	Average supplement consumption (g/day/animal)	Supplement P measured value (%DM)	Average phosphate intake (g/day/animal)†	% P consumed less than cattle fed supplement 3
Supplement 1	310	370	0.94	3.48	-47.7
Supplement 2	290	255	1.59	4.05	-16.0
Supplement 3	267	292	1.79	5.14	-

† , Calculated respective phosphate intake (grams per animal per day) to indicate the difference of phosphate intake between the respective groups.

### Lameness evaluation

The size of the peri-articular swelling and the degree of lameness of the trial cattle were used to determine the effect of the supplements. Visual grading of lesions has been statistically proven to be a valid method (unpublished data: K. Botha, 2016). Analysis of the initial values for all the cattle indicated that there were no significant differences pertaining to the sex, age, weight, lameness score, acute peri-articular swelling score, chronic peri-articular swelling (fibrosis) score and the overall disability score. The majority of cattle included in group 1 had acute peri-articular swellings, whereas the cattle included in groups 2 and 3 had very similar numbers of acute peri-articular swelling and chronic peri-articular swelling scores. Although the degree of lameness varied between animals within a group, the average lameness score ranged between 1 and 2 for all three groups.

Study results indicated no significant difference between the degree of lameness, acute peri-articular swelling, chronic peri-articular swelling and total disability score between the respective groups.

### Clinical examination and urinalysis

Four cattle from which urine could be collected were used for the clinical examination. None of the clinical parameters were abnormal for any of the four cattle examined. The urine of the respective animals was chemically evaluated with a urine test strip (Combur^9^Test^®^; Roche) and indicated a urinary pH of < 6 for all the four cattle.

### Statistical analysis

Statistical analysis indicated no significant difference between the respective supplements with regard to the measured parameters, with the exception of the acute peri-articular score of group 1 (Table 4). The significance thereof is, however, not of importance as the cattle only developed the more chronic form of the osteochondrotic peri-articular swelling (chronic peri-articular swelling).

**TABLE 4:** Statistical analysis of the baseline values as well as study results.

Parameters	Baseline values (*P*)†		Results (*P*)‡	
	
	Study 1	Study 2	Study 1	Study 2
Sex	0.774	0.384	-	-
Age	0.725	0.742	-	-
Weight	0.454	0.452	-	-
Lameness score	0.567	0.976	0.122	0.084
Acute peri-articular swelling	0.111	0.343	0.034§	0.247
Chronic peri-articular swelling	1.000	0.141	0.189	0.331
Overall disability	0.328	0.730	0.093	0.261

Significance based on a 95% confidence interval.

† , Based on chi-square tests for categorical variables and Kruskal–Wallis tests for quantitative data;

‡ , comparison among diets based on mixed-effects linear model analysing the change in variables from baseline (after rank transformation of scores) and adjusting for repeated measures by the addition of a random effect for animal;

§ , indicates significance.

## Discussion

The trial was conducted to determine the level of micro- and macrominerals required to be included in a supplement that would improve the clinical condition of cattle clinically affected by osteochondrosis.

The three supplements contained the same basic ingredients and only varied in mineral concentrations. The mineral levels of supplement 3 were based on those of the 2012 pre-trial supplement that had a significant positive effect when fed to cattle with clinical osteochondrosis (Prozesky et al. 2016).

The daily average supplement intake per animal between the groups varied (Table 3). Differing average starting weights (Table 3) and *ad lib* access to high quality *Eragrostis teff* hay would both have influenced the average daily consumption of supplements. *Eragrostis teff* hay is highly palatable and nutritious roughage that would contribute a large proportion of the nutritional daily requirements of the cattle. The most important factor was to calculate the mineral intake especially that of phosphate as it was thought that higher phosphate intake played a key role in the recovery and prevention of osteochondrotic lesions. Calculated average daily phosphate intake per animal differed among the respective groups. Group 3 had an average phosphate intake of 5.14 g per animal per day. Cattle of groups 1 and 2 consumed 47.7% (3.47 g) and 16% (4.05 g) less phosphate on a daily basis, respectively, when compared to the phosphate intake of group 3. It was expected that especially the cattle of group 3 would have responded positively to the higher micro- and macromineral intake, with group 1 responding the least to the supplementation.

Peri-articular swelling and lameness can occur simultaneously or independently of each other (Trostle et al. 1997, 1998). The size of the peri-articular swelling does not correlate with the extent of the osteo-arthritic lesion (unpublished data from radiographic analysis: Van der Veen), but is merely an indication of excessive synovial fluid produced as a result of joint inflammation (Trostle et al. 1998).

No work to date has been conducted to determine the time period it takes for the joint to deposit additional connective tissue in the capsule.

Baseline data and the final study results for groups 1, 2 and 3 indicated no significant difference between the lameness, acute peri-articular swelling, chronic peri-articular swelling and total disability scores.

All the cattle included in the study had clinical osteochondrotic lesions with the affected period of each animal unknown to the investigators. Several animals with acute peri-articular swellings developed chronic peri-articular swellings during the course of the study. The shift in chronicity of the peri-articular swelling is an indication that those animals did not respond to the supplementation but instead the severity of the osteochondrotic lesions remained the same or progressed.

It was expected that the cattle of this study fed supplement 3 would have responded positively in a similar fashion as did the cattle in the 2012 pre-trial, as the mineral levels included in supplement 3 resembled those of the pre-trial 2012 supplement.

The fact that none of the study cattle responded positively to any of the supplementations, regardless of different micro- and macromineral level intake between sub-groups, warranted further investigation. The results obtained in this study are contradictory to those observed during the 2012 pre-trial where cattle fed a supplement containing high levels of both micro- and macrominerals responded positively with a marked decrease in the severity of lameness and size of the peri-articular swelling (Prozesky et al. 2016).

Possible factors that could lead to the development of aciduria were investigated. It is known from studies in dairy cows that a dietary cation anion difference (DCAD) value of < -200 mEq/kg is effective in inducing a metabolic acidosis and aciduria (average pH 7) (DeGaris & Lean 2008).

The DCAD value for the respective trial supplements as well as the 2012 pre-trial supplement was calculated by subtracting the anion value from the cation value.

The most common equation used is [(Na^+^+ K^+^) - (Cl^-^ + S^2-^)] (DeGaris & Lean 2008). There was a significant difference between the DCAD value of supplements 1, 2 and 3 compared to that of the 2012 pre-trial supplement. Calculated DCAD values are provided in Table 5.

**TABLE 5:** Calculated dietary cation anion difference values of respective supplements.

Supplement	Calculated DCAD value (mEq/kg)†
Supplement 1 (Group1)	-411
Supplement 2 (Group 2)	-466
Supplement 3 (Group 3)	-467
2012 pre-trial supplement	+19.87

DCAD, dietary cation anion difference.

† , DCAD value calculated with the following equation: [(Na^+^+ K^+^) - (Cl^-^ + S^2-^)].

Further investigation into the composition of the trial supplements revealed that all three supplements contained the anionic salt, ammonium sulphate, whereas the pre-trial supplement did not contain an anionic salt, leading to a higher DCAD value.

A possible explanation why the elevated mineral intakes from supplements 2 and 3 did not result in recovery from osteochondrosis is as follows.

The plasma pH is regulated by four factors of which the strong ion difference is one. Intestinal absorption concentration of strong anions (chloride and sulphate) is more than that of strong cations (calcium, magnesium and ammonium) when salts like ammonium sulphate [(NH_4_)_2_SO_4_] are fed. The increased anion level in the plasma, in this case SO_4_^2-^, reduces the strong ion difference, inducing a strong ion metabolic acidosis (DeGaris & Lean 2008). Metabolic acidosis increases the responsiveness of tissue receptors to parathyroid hormone (PTH) (Horst et al. 1997). Bone responds to PTH by the activation of osteocytes as well as the osteoclasts (La Perle & Capen 2006) with osteoclastic activity increasing proportionally as the plasma becomes more acidic (Arnett 2003). Calcium can be mobilised from bone either in conjunction with or independently of PTH (DeGaris & Lean 2008). Bone acts as a buffer during acute metabolic acidosis by binding hydrogen ions to carbonate and releasing the cation salts (Na^+^, K^+^, Ca^2+^) associated with the carbonate into the extracellular fluid (Engelking 2011; Horst et al. 1997). This process, as well as the activation of osteoclasts through PTH, functions as a buffer during chronic metabolic acidosis (Green & Kleeman 1991).

Renal function responds to PTH through reduced reabsorption of phosphate (phosphaturia) and increased reabsorption of calcium (La Perle & Capen 2006) from the glomerular filtrate. Calcium is still excreted at elevated levels during metabolic acidosis despite the increased reabsorption action of PTH. The charge equivalence of albumin is altered during metabolic acidosis, which leads to the release of plasma protein bound calcium (up to 40% of the total calcium) increasing the amount filtered through the glomerulus of the kidney and ultimately excreted leading to increased mineral loss (Engelking 2011). The excess dietary anions in the plasma are filtered and excreted through the kidneys. The urine produced is acidic (aciduria) in nature because of the increased concentration of excreted anions (Spanghero 2004). A urinary pH < 5.5 indicates severe metabolic acidosis and should be avoided at all cost (Horst et al. 1997).

The majority of minerals reabsorbed from the skeleton of rats with metabolic acidosis occurred from the epiphysis, resulting in lower total bone volume of the metaphysis (Kraut et al. 1986). Mongin and Sauveur (1977) (q. Whitehead 1997) proposed that the anionic balance of a diet influenced the incidence rate of tibial dyschondroplasia (osteochondrosis) in chickens. Several studies followed that confirmed the proposal made by Mongin and Sauveur that metabolic acidosis increased the incidence of tibial dyschondroplasia in chickens (Whitehead 1997).

A new hypothesis was formulated based on the observations made during the trial. We hypothesise that chronic mild metabolic acidosis weakens the developing calcareous bone, consequently exposing particularly fast growing cattle to traumatic fracture of the subchondral bone and articular cartilage, leading to the development of lesions associated with osteochondrosis. This hypothesis also suggests that chronic metabolic acidosis will exacerbate subclinical or clinical osteochondrosis. These hypotheses require further investigation. It is important to consider not only the direct dietary cause of metabolic acidosis but to have a holistic approach which includes signalment, husbandry and regular analysis of both feed and water sources.

## Conclusion

Investigation of cattle clinically affected by osteochondrosis in the North West province on a herd basis revealed that bulls were more prone and severely affected than cows, with young animals more frequently affected than adult animals (Prozesky et al. 2016). The higher frequency among young animals is in accordance with the definition, as ‘osteochondrosis’ is a term that pertains to a group of lesions associated with the persistence of growth cartilage in the epiphyseal or physeal growth plates as a result of failure of endochondral ossification.

Prozesky et al. (2016) as well as data from the pre-trial (not shown) indicated that cattle clinically suffering from osteochondrosis responded positively when fed a supplement containing high levels of bio-available micro- and macrominerals.

The results from this study indicated that cattle with clinical osteochondrosis do not respond positively to a supplement with a low DCAD value, regardless of its micro- and macromineral concentration. The new hypothesis suggests that cattle supplemented for an extended period of time with a low DCAD feed are predisposed to the development of osteochondrosis or exacerbation of subclinical or clinical osteochondrosis. This hypothesis needs to be further investigated.
